# Molecular epidemiology of clinical *Staphylococcus aureus* infections: circulating CC398 lineage with human-adapted genomic signatures and virulent ST5-SCC*mec* II MRSA

**DOI:** 10.3389/fmicb.2026.1818812

**Published:** 2026-07-01

**Authors:** Qichen Wang, Chunjiao Liu, Tianzhu Liang, Jing Zhang, Yiwei Liu, Tianyin Zhou, Xiaojuan Jin, Jiaxing Yang, Daxi Wang, Ziqing Deng, Rong Zhang, Na Pei

**Affiliations:** 1 Department of Clinical Laboratory, Second Affiliated Hospital Zhejiang University, School of Medicine, Hangzhou, China; 2 BGI Research, Beijing, China; 3 Shenzhen Key Laboratory of Unknown Pathogen Identification, BGI Research, Shenzhen, China

**Keywords:** CC398, genomic epidemiology, small colony variant, ST5, *Staphylococcus aureus*

## Abstract

Clinical *Staphylococcus aureus* infections, such as pneumonia and bloodstream infections, pose significant threats and potentially lead to fatal outcomes. Most clinical isolates harbor multiple virulence factors and antimicrobial resistance genes. This study focused on *S. aureus* strains isolated from clinically documented infections at a Chinese tertiary hospital between 2018 and 2023. Using whole-genome sequencing, we characterized the genomic epidemiology of 262 strains. In addition, long-read sequencing and Bayesian analysis were used to characterize the genomic features associated with clonal distribution of *S. aureus* isolates. We identified ST239-III, ST398-V, ST59-IV, and ST5-II as the predominant methicillin-resistant *S. aureus* (MRSA) clones, which belong to clonal complexes CC5, CC8, CC59, and CC398. Majority of CC5 strains were distinguished by their carriage of the toxic shock syndrome toxin-1 gene *tst-1*, phiN315-like *hlb*-converting prophages, and type II SCC*mec*. Notably, most *tst-1*-positive ST5-MRSA-SCC*mec* II isolates exhibited a small colony variant phenotype with reduced growth rate, attenuated hemolytic activity, and enhanced biofilm formation, suggesting a potential role in persistent infections. In contrast, CC8 strains were prevalent due to the typical multidrug-resistant profile of ST239 lineage. Interestingly, the dominant CC398 isolates belonged to a human-adapted clade, underscoring its clinical relevance in nosocomial settings. These results showed CC5, CC8, and CC398 as the dominant clones in clinical isolates and offered preliminary genomic observations on their adaptive and pathogenic-related characteristics.

## Introduction

*Staphylococcus aureus* is one of the leading pathogens causing a range of infections from mild skin and soft tissue infections to severe and life-threatening diseases, such as pneumonia, bacteremia, sepsis, endocarditis, and toxic shock syndrome (TSS) (Lakhundi and Zhang, 2018). Clinical *S. aureus* infections often originate from localized superficial sites or are associated with prosthetic devices or indwelling catheters. Epidemiological data from the China Antimicrobial Surveillance Network (CHINET, http://www.chinets.com/Data/AntibioticDrugFast) and Global Burden of Diseases study 2019 underscored its significant burden, ranking *S. aureus* as the third most prevalent pathogen (Zhang et al., 2019; Ikuta et al., 2022). A 12-year study in the United States identified two sequence types (STs) (ST5 and ST8) as the predominant clones in bloodstream infections and causing majority of methicillin-resistant *S. aureus* (MRSA) infections (Souza et al., 2024). This is in contrast to the epidemiological landscape in China, where ST59, which is a common CA-MRSA clone, and ST239, which is a predominant HA-MRSA clone, were reported to be more widespread (Zhou et al., 2025). Given the dynamic nature of *S. aureus* population structures and the potential for clonal shifts worldwide, continuous genomic surveillance remains essential to understanding the evolution and global dissemination of this bacterium.

The success of *S. aureus* as a formidable pathogen stems from its expression of diverse virulence determinants and the escalating problem of antimicrobial resistance. The pathogenicity of *S. aureus* is largely driven by its production of a wide range of virulence factors, including surface adhesion proteins for host cell attachment, and extracellular toxins that cause direct host cell damage and immune dysregulation. Key toxins include hemolysins, which are encoded by genes, such as *hla, hlb, hld,* and *hlg*; Staphylococcus enterotoxins (SE), which are encoded by genes, such as *sea* to *sei*; Panton–Valentine leukocidin (PVL), which is encoded by *lukS/F-PV*; and toxic shock syndrome toxin-1 (TSST-1), which is encoded by *tst-1* and can trigger the life-threatening systemic illness TSS (Lowy, 1998; Liu et al., 2024; Taki et al., 2022). The *tst-1* gene is typically located on the *S. aureus* pathogenicity island (SaPI), which can also carry the SE genes of *sec* and *sel*, particularly in CC5 (Zhao et al., 2019). Strains that coproduce TSST-1 and SEs have been associated with fatal outcomes (Taki et al., 2022). Furthermore, Staphylococcus enterotoxin A (*sea*) is frequently located on a *β*-hemolysin-converting bacteriophage together with the genes *sak* (encodes staphylokinase), *chp* (encodes the chemotaxis inhibitory protein of *S. aureus*), and *scn* (encodes staphylococcal complement inhibitor) to form a defined immune evasion cluster (IEC). Phage-encoded IEC can block complement C3b formation on the bacterial surface and inhibit the capability of host neutrophils to phagocytose *S. aureus* (van Wamel et al., 2006; Rohmer and Wolz, 2021). The presence of type B IEC, which contains *sak*, *chp*, and *scn*, and absence of *tetM* serve as a marker for the human-adapted clade of CC398 (Fernandez et al., 2024). The human adaptation of CC398 clade is marked by acquisition of IEC, which enhances host colonization, and concomitant loss of the tetracycline resistance gene *tetM*. This genetic signature suggests an adaptive trajectory that is distinct from animal-associated strains and is driven by different antibiotic selection pressures and the necessities of immune evasion in the human host. Taken together, the pathogenic behavior of *S. aureus* is closely linked with the distribution and expression of its virulence genes, which vary among genetic backgrounds and ecological niches.

In addition to its virulence, *S. aureus* poses a serious threat due to its broad antibiotic resistance, which severely limits treatment options (Lee et al., 2018). The emergence of MRSA has been largely attributed to the acquisition of the *mecA* gene, which is located on the Staphylococcal cassette chromosome *mec* (SCC*mec*) elements, which confer resistance to nearly all *β*-lactam antibiotics (Liu et al., 2016; Turner et al., 2019). In China, molecular epidemiological studies have identified CC8-ST239, CC59-ST59, and CC5-ST5 as the predominant MRSA clones, with their prevalence characterized by dynamic shifts over time (Hao and Wang, 2025). Therefore, accurate characterization of circulating *S. aureus* clones in healthcare settings, along with identification of the drivers behind dominant lineages, is essential for effective and timely infection control.

In this study, we comprehensively analyzed 262 *S. aureus* isolates from clinical infections by genomic epidemiology and characterized the genomic and phenotypic features of dominant clones to identify lineage-associated traits that are linked with clonal prevalence.

## Materials and methods

### Ethics approval

This retrospective study was approved by the ethics committee of the Second Affiliated Hospital of Zhejiang University School of Medicine (number 2024-1037). Informed patient consent was waived, because samples were taken routinely under a hospital surveillance framework. The research conformed to the Principles of the Helsinki Declaration.

### Source of strains and information

All consecutive nonduplicate *S. aureus* isolates (total of 262 strains) from clinically documented infections were collected at the Second Affiliated Hospital of Zhejiang University between December 2018 and July 2023. The sample sources included blood, sputum, bronchoalveolar lavage fluid (BALF), urine, and abscess (Supplementary Table 1). Only strains from patients with clinically and microbiologically confirmed infection were analyzed. The criteria for infection included fever, leukocytosis, elevated CRP/PCT, radiological infiltrates, or local purulent manifestations. Microbiological criteria were applied according to the specimen type: (1) at least one positive blood culture with a typical pathogen consistent with clinical infection, (2) BALF culture consistent with lower respiratory tract infection plus radiological evidence, (3) sputum of acceptable quality with predominant pathogen growth plus radiological evidence, (4) significant bacteriuria (≥10^5^ CFU/mL) with clinical symptoms, and (5) abscess containing purulent material with predominant pathogenic growth. Isolates that represented colonization or contamination were excluded.

### Strain identification

Isolates were identified at the species level using matrix-assisted laser desorption ionization–time of flight mass spectrometry (Bruker Daltonik GmbH, Bremen, Germany).

### Antimicrobial susceptibility testing

Antimicrobial susceptibility testing was performed using an automated bacterial identification and susceptibility system (Vitek 2 Compact, bioMérieux, France), according to the Clinical and Laboratory Standard Institute guidelines (CLSI, 2024). For disk diffusion tests, *S. aureus* ATCC 25923 was used as the quality control strain, while ATCC 29213 was adopted for Vitek 2 assays. The suspension was prepared at a 0.5 McFarland standard concentration, which was approximately 1.5 × 10^8^ CFU/mL. The Vitek 2 AST-GP67 (catalog no: 22226) susceptibility card was used, and the tested antibiotics included cefoxitin (screen), oxacillin, penicillin G, gentamicin, rifampicin, ciprofloxacin, levofloxacin, clindamycin, trimethoprim/sulfamethoxazole, moxifloxacin, linezolid, vancomycin, quinupristin/dalfopristin, tetracycline, tigecycline, and erythromycin. According to CLSI guidelines, cefoxitin and oxacillin results were used for MRSA identification and *β*-lactam susceptibility interpretation.

### DNA extraction and whole-genome sequencing

DNA was extracted using the QIAGEN Midi Kit (Qiagen, Hilden, Germany), according to the manufacturer’s protocol, with the addition of lysostaphin (Sigma-Aldrich, Missouri, United States) during the bacterial cell lysis procedure. For all 262 isolates included in this study, whole-genome sequencing (WGS) was performed on a short-read sequencing platform (DNBSEQ-T5, MGI, Shenzhen, China) using the 150 bp paired-end libraries prepared by MGIEasy Universal DNA Library Prep Set (MGI, Shenzhen, China). Furthermore, representative MRSA isolates that covered major STs were prioritized for long-read WGS. Strains with inconsistent genotypic and phenotypic characteristics were also selectively included. In total, 26 isolates were selected for long-read sequencing (CycloneSEQ-WT02, MGI, Shenzhen, China) in order to reflect the genetic diversity, phenotypic variation, and dominant lineage features of the clinical *S. aureus* isolates in this study. The libraries were prepared using the H940-000018 CycloneSEQ 24 Barcode Library Prep Set and H940-000016 CycloneSEQ WT Sequencing Kit (6 T).

### Quality control of WGS data

The raw read number of the PE150 sequencing data was approximately 36 million reads per sample and were trimmed and qualified by fastp version 0.14.0 (Chen et al., 2018). MetaPhlAn3 was used to confirm the species of *S. aureus* (Beghini et al., 2021). For the long-read sequencing data, Porechop version 0.2.4 and NanoFilt version 2.8.0 were used for adapter removal and data quality control (Wick et al., 2017; De Coster et al., 2018).

### *De novo* assembly and genome annotation

SPAdes version 3.10.0 was used for the *de novo* assemblies using the PE150 data with k-mer sizes of 55, 77, and 99 (Bankevich et al., 2012). The assembled contigs were filtered and kept as follows: contig lengths were >500 bp, GC contents were between 32 and 33%, and genome lengths were 2.5–3.1 Mb. Unicycle version 0.5.1 was applied for the hybrid assembly using both the PE150 data and CycloneSEQ long-read sequencing reads (Phillippy et al., 2017). Prokka version 1.13 was used for gene annotation (Seemann, 2014).

### MLST, SCC*mec* typing, and *spa* typing

The assembled contigs were analyzed using MLST version 2.23.0^1^ (Jolley et al., 2018). Clone complex (CC) was grouped based on the allele differences in one or more housekeeping genes.^2^ SCC*mec* typing was performed using the SCC*mec*Finder (Kaya et al., 2018) at the Center for Genomic Epidemiology, Technical University of Denmark.^3^ The *spa* typing was done by spa_typing.^4^

### Analysis of resistance and virulence genes

Antimicrobial-resistant genes (ARGs) were identified using ResFinder version 4.0 (Bortolaia et al., 2020). Virulence factors (VFs) were identified using a virulence factor database (updated on July 28, 2019). Genes with >80% identities and >80% coverages were identified.

### Pangenome analysis and principal component analysis

Pangenome analysis of all 262 *S. aureus* isolates was performed using Roary v3.13.0 with parameters -e --mafft. Annotated GFF files generated by Prokka v1.13 were used as input files. The resulting gene presence–absence matrix was adopted for subsequent principal component analysis (PCA). PCA was conducted in R v4.2.2 using the fast.prcomp() function of the gmodels package (v2.18.1), and PCA visualization was generated using the ggpubr package.

### Phylogenetic tree construction

All the 262 WGS samples were aligned by high-quality reads mapping to the complete genome of the *S. aureus* strain N315 (BA000018.3). Core genome SNPs were identified by snippy-core and filtered using a minimal read depth of 10 × and a minimal variant allele frequency of 90% using snippy version 3.2.^5^ Phylogenetic trees were constructed using IQ-TREE, with the parameters -bb 1,000 -nt 8 -seed 1 -m GTR + I + G (Nguyen et al., 2015). Visualization was implemented on iTOL.^6^

### Public genome collection and filtering criteria

All publicly available genomes downloaded from National Center for Biotechnology Information (NCBI) and PubMLST were subjected to uniform quality and metadata filtering. Only high-quality genomes with complete assembly information, clear collection year, geographic location, and isolation host were retained; low-quality draft genomes and genetically redundant strains were excluded to reduce sampling bias.

For CC398, a total of 100 qualified public genomes were selected to cover distinct geographic regions (Asia, Europe, and North America) and host origins (human, livestock, and environment) with different collection years. For ST5, strains with complete metadata on collection date, continent, country, and isolation source were first screened from the PubMLST *Staphylococcus aureus* database. Additional high-quality ST5 genomes were retrieved from NCBI, according to three published studies that focused on global and Asian ST5 genomic epidemiology. After merging datasets, duplicate and closely related strains were removed. Only *tst-1*-positive isolates were retained, yielding a final dataset of 325 genomes, including our in-house isolates, for subsequent BEAST analysis.

### Time-calibrated phylogeny

Timed phylogenetic trees for the 138 CC398 isolates and 325 *tst-1*-positive ST5 isolates were reconstructed using BEAST2 (Bouckaert et al., 2019). We obtained 100 CC398 genomes and 269 *tst-1*-positive ST5 genomes from the NCBI public database and 40 *tst-1*-positive ST5 genomes from PubMLST (Supplementary Tables 2, 3). All contigs from the 138 CC398 isolates were mapped to the ST398 reference genome (AM990992.1), whereas those from the 325 ST5 isolates were mapped to the N315 reference strain (BA000018.3). XML configuration files were generated using BEAUti2 with dataset-specific model settings. For the CC398 dataset, we applied the GTR substitution model, a strict molecular clock, and a coalescent constant population prior. The Markov Chain Monte Carlo (MCMC) analysis was run for 30 million steps, sampling every 10,000 steps with a 10% burn-in. For the ST5 dataset, the TN93 substitution model was used along with a relaxed exponential molecular clock and a coalescent Bayesian Skyline prior. The MCMC was set to 200 million steps, sampling every 10,000 steps and discarding the first 10% as burn-in. All BEAST2 outputs were summarized using TreeAnnotator v2.7.1 to generate maximum clade credibility trees for visualization and downstream analyses. Prior to BEAST analysis, temporal signals were assessed using TempEst v1.5.1, and a clear positive correlation was identified in both CC398 and ST5 datasets, confirming adequate temporal structure for molecular clock calibration. MCMC convergence was evaluated using Tracer v1.7.2; all parameters yielded effective sample sizes (ESS) > 200, indicating sufficient mixing and reliable convergence.

### Lysis of erythrocytes by culture filtrates

We collected 100 μL of the BHI overnight culture supernatants of the six *S. aureus* ST5 strains, including three randomly selected *tst-1*-negative strains of SA56, SA157, and SA231, from different years in blood and three CycloneSEQ strains carrying *tst-1*; these were incubated with 100 μL of 2% human red blood cells in 0.9% saline for 1 h at 37 °C. Hemolytic activity was measured by testing the optical density values at 540 nm (OD540) twice using a Multiskan FC Microplate Photometer (Thermo Scientific).

### Bacterial growth curve for the ST5 isolates

A total of 24 ST5 strains, including 12 representative *tst-1*-negative and 12 *tst-1*-positive ST5 isolates, were selected. Our collection included 16 *tst-1*-positive and 14 *tst-1*-negative ST5 isolates recovered between 2020 and 2023. All viable strains from this period were included, and equal sample sizes of 12 isolates per group was chosen to ensure balance and comparability. Notably, all selected 12 *tst-*1-positive ST5 isolates were MRSA, whereas all 12 *tst*-1-negative ST5 isolates were MSSA. This grouping corresponded to the natural distribution of methicillin resistance and *tst*-1 carriage among the ST5 isolates in our hospital cohort. *S. aureus* isolates were grown overnight in 5 mL of Luria–Bertani (LB) lysogeny broth at 37 °C then centrifuged (200 rpm) (Supplementary Table 4). Overnight cultures were diluted 1:1000 in 200 μL of fresh LB broth then centrifuged (200 rpm) before placing back to the incubator at 37 °C. The OD600 of the final cultures were monitored automatically every hour using a Multiskan FC Microplate Photometer (Thermo Scientific). The assay was performed in triplicate, and the mean values were used to draw the figure.

### Semiquantitative biofilm assay

Each overnight culture of the 24 ST5 strains were diluted 1:500 in fresh LB broth, then were pipetted into 96-well flat-bottom tissue culture plates and incubated at 37 °C for 24 h without centrifuge. After gently removing the culture supernatants, the wells were washed twice with 0.9% saline, and adherent biofilm was placed in Bouin’s fixative for over 1 h. The fixative was removed gently, and saline was used to wash each well twice. Subsequently, the biofilm was stained with crystal violet, and the floating stain was washed away with slow-running water. After drying at room temperature, thickness of the stained biofilm was quantified using a Multiskan FC Microplate Photometer (Thermo Scientific) at 570 nm. The assay was performed in triplicate, and the mean values were used to draw the plot.

### Statistical analyses

Statistical analyses were performed using R version 3.6.1. Differences in prevalence between STs and CCs were analyzed using Kruskal–Wallis test. Heatmap was made using the pheatmap package. Sanki diagram was drawn using Python. Nonparametric tests were performed, with *p* < 0.05 considered as statistically significant.

## Results

### Molecular epidemiology and genomic characterization of the 262 clinical *Staphylococcus aureus* in China

**Figure 1:**
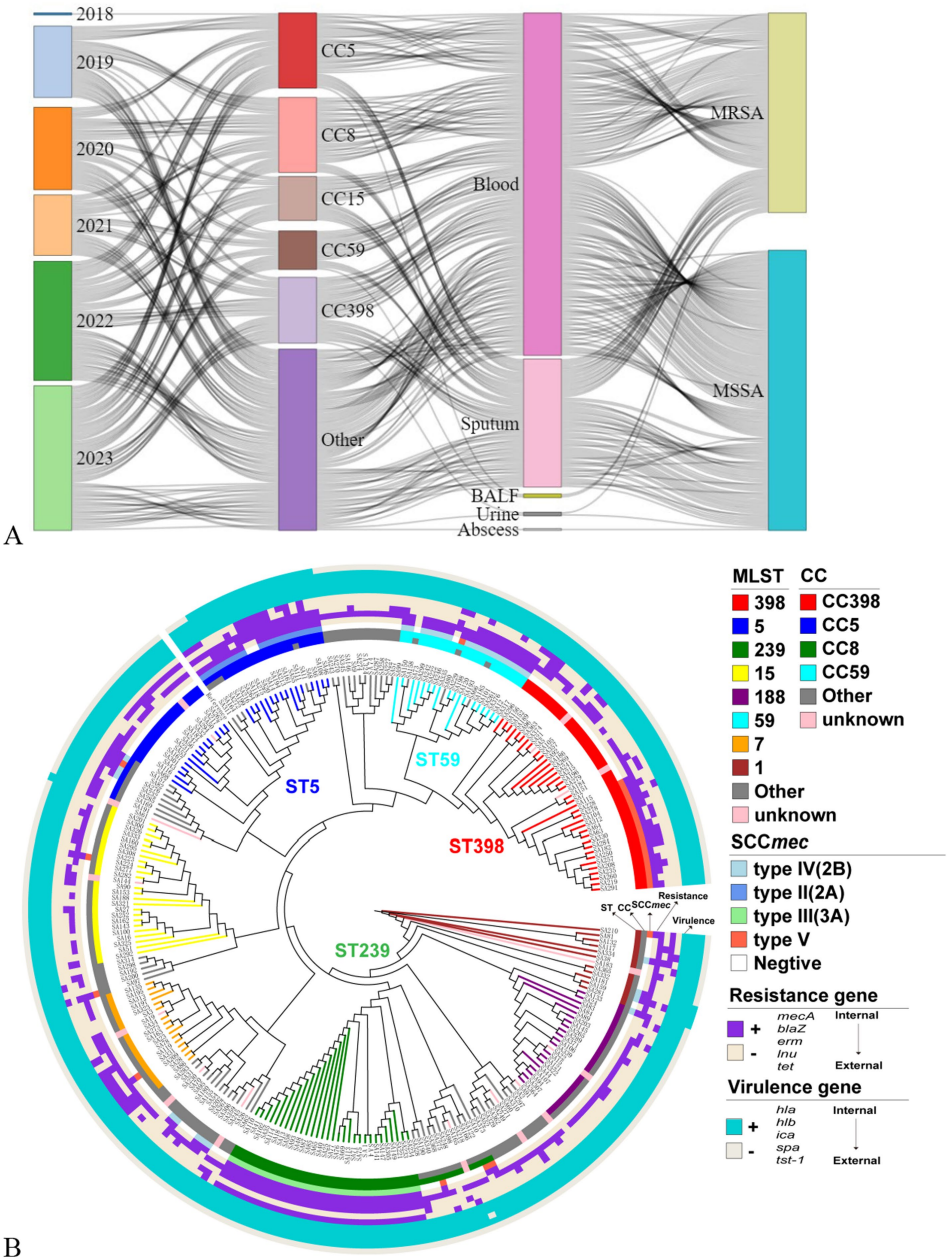
Characteristics of the *S. aureus* isolates in this study. **(A)** Sankey diagram depicting the relationships among the isolation year, major clonal complexes (CCs with >20 strains), sample sources, and methicillin (oxacillin) susceptibility for the 262 strains. **(B)** Core genome maximum likelihood phylogenetic tree of the 262 strains. Branch colors and the innermost ring represent the MLST types. The subsequent rings from the inside out display the clonal complex (CC) groups; SCC*mec* types; ARGs of *mecA*, *blaZ*, *erm*, *lnu* and *tet*; and virulence factors of *hla*, *hlb*, *ica*, *spa*, and *tst-1*.

MLST analysis divided the 262 strains into 25 known types (*n =* 249) and currently unknown types (*n =* 13, database version October 28, 2022, latest access on January 17, 2024) (Supplementary Table 1). The most prevalent MLST types were ST398 (*n =* 36, 13.7%), followed by ST5 (*n =* 30, 11.5%) and ST239 (*n =* 30, 11.5%). CC analysis further grouped the strain types into 17 known CCs, with the largest being CC5 and CC8 (*n =* 41, 15.6%), followed by CC398 (*n =* 36, 13.7%) and CC59 (*n =* 21, 8.0%) (Supplementary Figure 1). Notably, CC398 showed an increasing trend from 5% in 2019 to 20% in 2023 (Figure 1A). In contrast, ST239, which is historically a dominant multidrug-resistant clone in China, exhibited a gradual decline in relative prevalence over the study period (Supplementary Figure 6). The distribution of sources of clinical *S. aureus* isolates differed significantly among the four predominant STs (ST5, ST239, ST398, and ST59). Chi-square test confirmed a significant difference in infection site distribution among these STs (*p* < 0.05). ST239 was strongly associated with bloodstream infection (29/30, 96.7%), with only one isolate recovered from the respiratory tract. Most of the ST398 isolates (*n* = 23, 63.9%) in our collection were from blood specimens; nevertheless, this lineage remained the most frequently isolated from respiratory specimens (12/36, 33.3%), compared with the other STs. ST5 and ST59 showed relatively moderate distributions without pronounced site predilection.

Of the 262 strains, 100 (38.2%) were identified as MRSA by AST; 77 were from bloodstream infections, 22 were from respiratory samples, and 1 was from a urine sample. Meanwhile, the *mec* gene was detected in 104 genomes (39.7%); only *mecA* was detected, and no *mecC* was found. We identified three *mecA*-negative MRSA, and six were *mecA*-positive MSSA. SCC*mec* typing was performed on the 104 *mecA*-positive isolates. There were 18 type II (2A) SCC*mec* elements identified in CC5 (14 ST5, 3 ST764, and 1 ST5530); 30 type III (3A) SCC*mec* elements in CC8 (ST239); 30 type IV (2B) SCC*mec* elements in 18 CC59 (17 ST59 and 1 ST3355); 3 CC/ST1, 3 CC5/ST965, 3 CC/ST88, 2 CC/ST6, and 1 CC/ST188 isolates; and 26 NGS nontypable but CycloneSEQ-typable type V SCC*mec* elements (Supplementary Figure 2). The type V SCC*mec* variants were mainly distributed in CC/ST398 (*n* = 17, 65.4%), followed by CC8 (ST630, *n =* 3, 11.5%); CC/ST1 (*n =* 1, 3.8%); CC5 (ST965); CC/ST7; CC/ST15; CC59 (ST338); and CC/ST72. In summary, the most common MRSA clones were ST239-III (3A) (*n =* 30, 11.5%), ST398-V (*n =* 17, 6.5%), ST59-IV (2B) (*n =* 17, 6.5%), and ST5-II (2A) (*n =* 14, 5.3%).

To fully characterize the genomes of our strain collection, pangenome analysis identified a total of 10,288 genes, including 1,885 core genes (≥95% shared) and 8,403 accessory genes. PCA effectively discriminated the MLST groups and CC clusters, with CC/ST398 and CC8 (ST239) notably separated from the main clusters (Supplementary Figures 3A,B).

The ARGs and VFs of the 262 strains on core genome maximum likelihood (ML) phylogenetic tree are shown in Figure 1B. A total of 80 VFs and 29 ARGs were identified (Supplementary Table 1; Supplementary Figure 4). The TSS toxin-1 (TSST-1) encoding gene *tst-1* was identified in 21 (8%), and 81% (*n =* 17) were carried by the largest CC5 clone; only 3 in CC/ST1 and 1 in a strain of SA61, a new MLST type. Moreover, in CC5, more than half (22/41) of the isolates carried *mecA* and almost all (18/22) harbored type II (2A) SCC*mec* elements. Paired-end 100 bp (PE150) sequencing data showed that one SCC*mec* element carried by the ST965 strain SA82 remained nontypable. Therefore, CycloneSEQ was performed for SA82, and the SCC*mec* element was classified as type V, showing the power of long-read sequencing (Supplementary Figure 2).

CC8 mainly comprised ST239 isolates (30/41, 73.2%). The ST239 isolates that were clustered together in the ML tree and had the greatest number of ARGs identified included *mecA* for methicillin resistance; *aac(6′)-aph(2″)*, *ant(9)-Ia*, and *aph(3′)-III* for aminoglycoside resistance; *blaZ* for penicillin resistance; *dfrG* for trimethoprim resistance; *erm(A)* for macrolide, lincosamide, and streptogramin B resistance (MLS_B_ phenotype); and *tetM* for tetracycline resistance (*n =* 30, 100%). These results showed a highly consistent resistance pattern, suggesting the superiority of ST239-specific multiple drug-resistance (MDR) gene pattern. The MDR gene patterns included the following: (1) type III (3A) SCC*mec* element, (2) IS*256*-*aac(6′)-aph(2″)*-IS*256*-*aph(3′)-III*-*ant(6)-Ia*, (3) *erm(A)*-*ant(9)-Ia*-Tn*554*, (4) integrase-*tet(M)*-*dfrG*, and (5) Tn*552*-*blaZ*.

### The increased CC398 belonged to the global human-adapted clade

**Figure 2:**
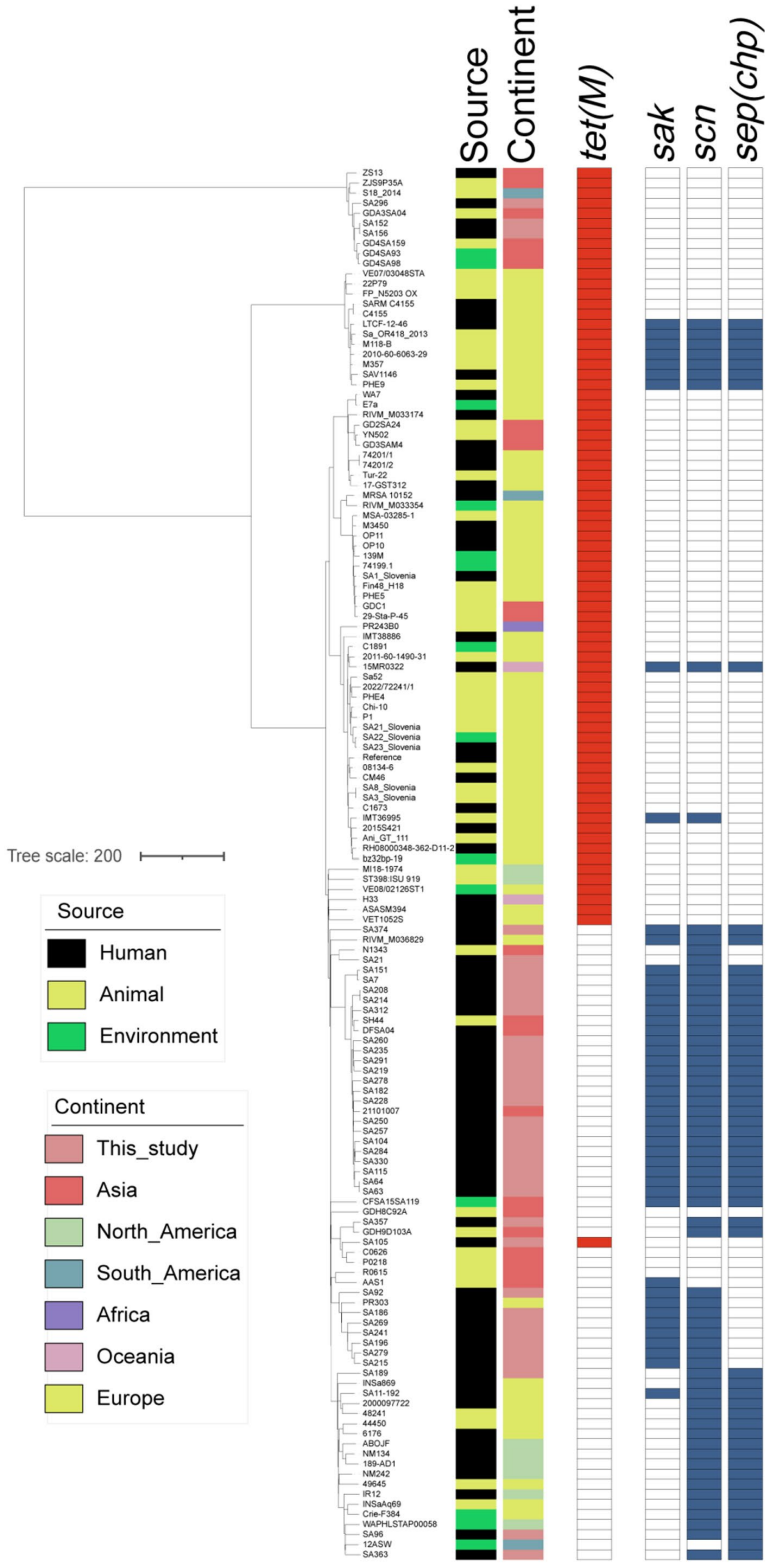
Time-calibrated phylogenetic tree of a global collection of 138 *S. aureus* CC398 genomes.

The prevalence of CC398 in this study showed an increasing trend, compared with the others (Supplementary Figure 5). Moreover, these CC398 isolates exhibited a distinctive profile of lacking *tetM* but harboring *sak*-*chp*-*scn*-encoding type B IEC, which is a hallmark of the human-adapted clade of CC398 (Figure 1B). To see if these CC398 isolates clustered together with the human-adapted clade and aligned with the evolution timeline of global CC398, we randomly retrieved 100 publicly available CC398 genomes from GenBank and constructed a time-calibrated phylogenetic tree using a total of 138 genomes (Figure 2; Supplementary Table 2). The CC398 isolates were distinctly divided into three principal clusters. Majority of these isolates were clustered together with human-associated isolates from Asia. Consistent with this phylogenetic division, the accessory gene repertoire further separated the livestock and environmental isolates from the human-adapted CC398 clade, underscoring distinct evolutionary paths.

The tree includes isolates from human, animal, and environmental sources, with tip labels showing sample names (this study) and public database accession/BioProject numbers. The presence of the *tetM* and immune evasion cluster genes [*sak*, *scn*, and *sep* (*chp*)] is indicated.

### Morphology variations between *tst-1*-positive and *tst-1*-negative ST5 isolates

**Figure 3:**
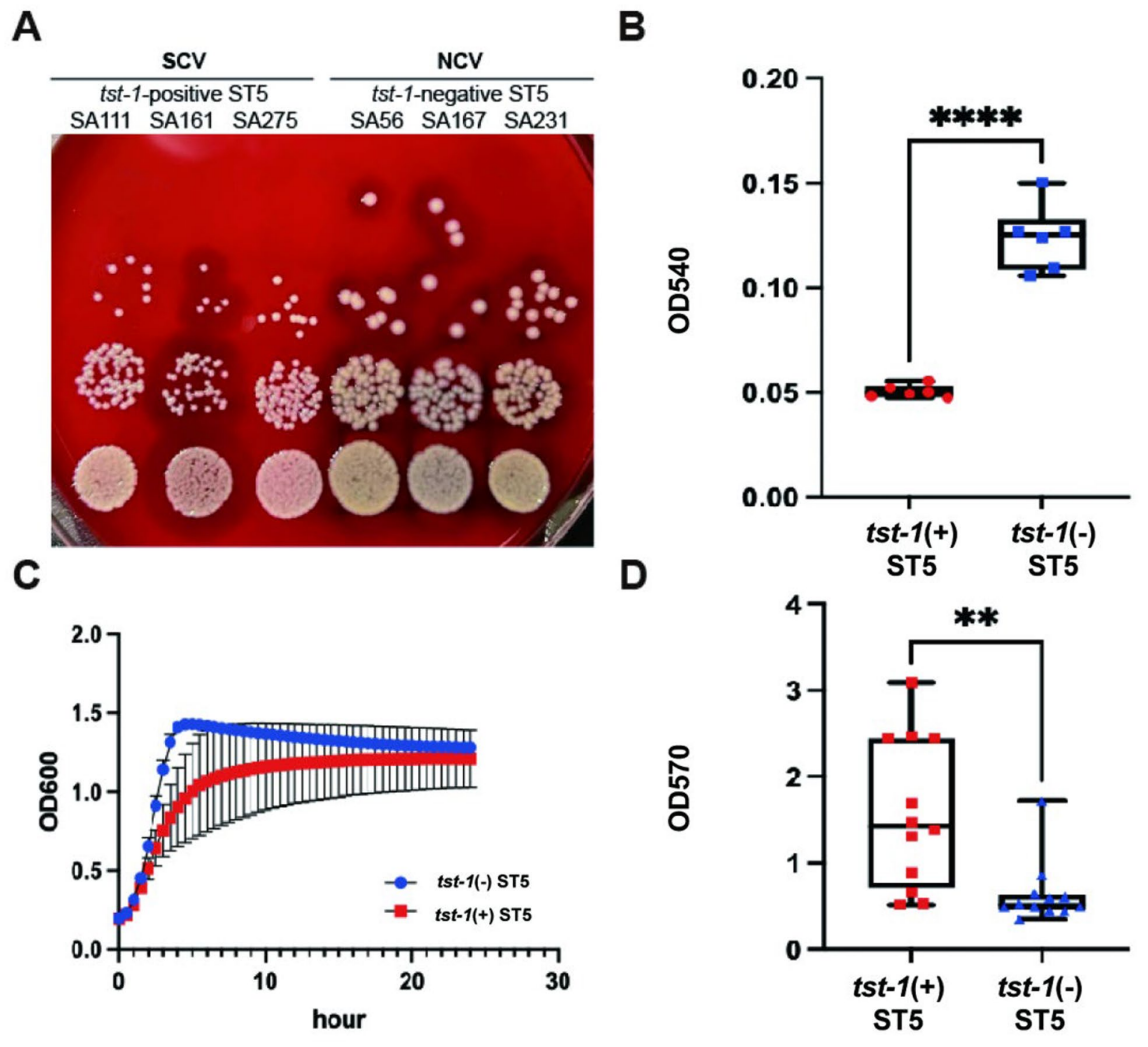
Altered morphology differences between the representative *tst-1*-positive and *tst-1*-negative ST5 isolates. **(A)** Difference in colony morphology among six representative isolates on blood agar; the isolates (*tst-1*-negative ST5: SA56, SA167, and SA231; *tst-1*-positive ST5: SA111, SA161, and SA275) were diluted in 1.5 × 10^7^ CFU/mL, 1.5 × 10^6^ CFU/mL, 1.5 × 10^5^ CFU/mL, and 1.5 × 10^4^ CFU/mL. **(B)** Difference in erythrocyte lysis capacity (t = 11.224, df = 10, *p* < 0.0001). **(C)** Growth curves and **(D)** semiquantitative biofilm formation capabilities (t = 3.442, df = 22, *p* = 0.0023) of ST5 representative isolates that were *tst-1*-negative (*n* = 12) and *tst-1*-positive (*n* = 12). Only the mean values from the triplicate tests for each strain were depicted as dots. Data are presented as mean ± standard deviation. ** *p* < 0.01, ****p < 0.0001.

Variations in morphology, including colony size and hemolytic characteristics on blood agar, were observed in the ST5 strain cultures (Figure 3A; Supplementary Table 3). Morphology differences were highly associated with *tst-1* gene carriage (chi-square, *p* < 0.001) (Table 1). Of the 12 *tst-1*-positive ST5 isolates, 75% showed small colony variant, and more than half (6/9) did not show hemolysis. In contrast, all 12 *tst-1*-negative ST5 isolates showed normal colony variant with strong hemolytic capabilities. In addition, differences in erythrocyte lysis capability were confirmed by optical density measurement at 540 nm of the 4-h incubation test on two replicates (*p* < 0.0001) (Figure 3B). Compared with the *tst-1*-negative ST5 isolates, the *tst-1*-positive ST5 isolates had slower growth rates (Figure 3C) and higher biofilm formation capability (OD570 1.575 ± 0.867 vs. 0.640 ± 0.365, *p* = 0.0023) (Figure 3D). Notably, 6 of 12 *tst-1*-positive ST5 isolates were obtained from patients clinically diagnosed with TSS. In summary, the ST5-II (2A) isolates positive for *tst-1* presented with small colony sizes, diminished capacity for erythrocyte lysis, slow growth rates, and relatively high biofilm formation capabilities.

**Table 1:** Morphology differences between the *tst-1*-positive and *tst-1*-negative ST5 isolates.

Hemolytic phenotype	ST5 *tst-1*-positive (*n =* 12)		ST5 *tst-1*-negative (*n =* 12)	
	SCV	NCV	SCV	NCV
No hemolysis	6 (50%)	0	0	0
Weak hemolysis	3 (25%)	3 (25%)	0	0
Hemolysis	0	0	0	12 (100%)

SCV, small colony variant; NCV, normal colony variant.

### Integration of phiN315-like *hlb*-converting phages into *tst-1*-carrying ST5 MRSA isolates inhibited hemolysis

**Figure 4:**
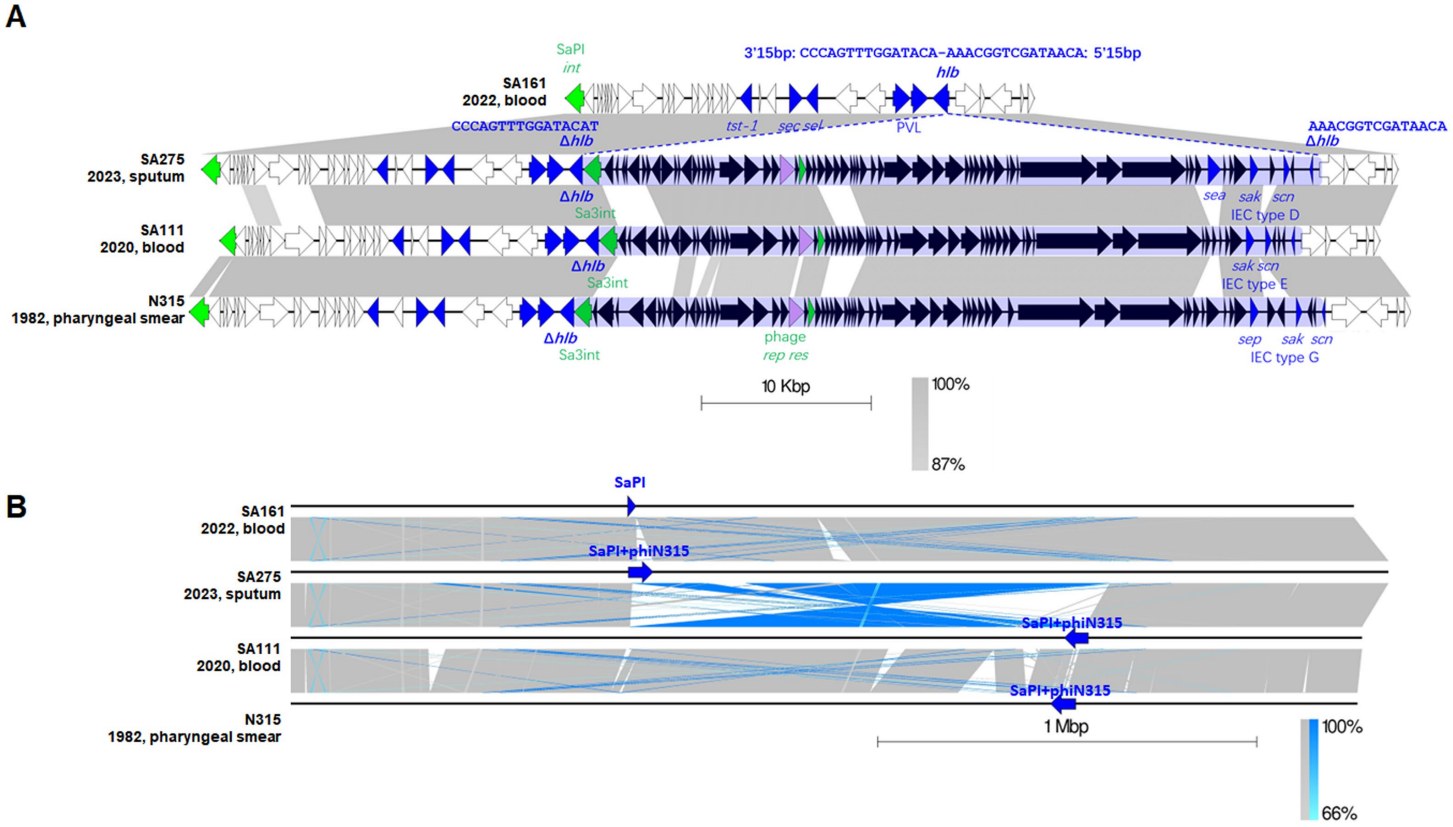
Genomic alignment of the *tst-1*-positive ST5-II (2A) *S. aureus* isolates. **(A)** Sequence alignment shows the *tst-1*-carrying enterotoxin gene island SaPIs and the ICE-carrying *hlb*-converting prophages. The PVL genes in these ST5-II MRSA isolates are located within an N315-like hlb-associated prophage, which is distinct from canonical *φ*-PVL prophages and not on the SaPI element. The areas in gray illustrate the nucleotide similarities and genetic relatedness. Green, blue, and black arrows and areas in blue denote the mobile genetic elements, virulence factors, phiN315-like *hlb*-converting phages, respectively. Portions of the *hlb* gene sequences are also displayed. **(B)** Chromosomal alignment and gene locations of the *tst-1*-carrying SaPIs and ICE-carrying phiN315-like phages. The shadings illustrated the nucleotide similarities and genetic relatedness.

To better understand morphology differences and elucidate the mechanisms of *tst-1* carriage in ST5, we sequenced three *tst-1*-carrying ST5-II (2A) isolates (SA111, SA161, and SA275) using CycloneSEQ. Following the hybrid assembly, we obtained complete genomic sequences and pinpointed the gene locations of *tst-1* on SaPIs (Figure 4A). The *tst-1* harboring SaPI contained more VFs, such as *sec* and *sel*, which encode staphylococcal enterotoxins, and *lukS/F-*PV, which encodes the PVL toxin. Compared with the canonical *φ*-PVL prophage that typically carries PVL genes, PVL was found to be integrated in an N315-like *hlb-*associated prophage in our ST5-II MRSA isolates. BLAST comparison with phiPVL-CN125, phiPV83, phiPVL108, and phi2958PVL confirmed low sequence similarity, supporting that these PVL genes were not part of the typical φ-PVL elements. Furthermore, the PVL genes were distinct from SaPI elements in these strains. More intriguingly, an *hlb*-converting phage was identified next to the SaPI harboring *tst-1* in both nonhemolytic *tst-1*-positive ST5-II (2A) isolates, SA111 and SA275; this explained the nonhemolytic properties of these *tst-1*-positive ST5-II (2A) isolates. Notably, the IEC type carried by these *hlb*-converting phages differed among isolates. In SA275 isolated from sputum in 2023, there was a type D IEC containing the genes *sea*, *sak*, and *scn*, which encoded enterotoxin A, staphylokinase, and staphylococcal complement inhibitor, respectively. While, in SA111 isolated from blood in 2020, there was a type E IEC comprising only *sak* and *scn* genes. In the reference N315 genome, the IEC was type G and contained the genes *sep*, *sak*, and *scn*.

More interestingly, compared with the earlier strains of N315 and SA111, SA161 and SA275 demonstrated a large-scale (approximately 1 Mb) chromosomal flip-flop inversion. Notably, *tst-1* carrying SaPI and the *hlb*-converting phage were found in close proximity with the breakpoints of chromosomal variation (Figure 4B). This finding provided intriguing insights into the genetic plasticity and adaptability of these *tst-1*-positive ST5-II (2A) strains. However, no significant morphology differences were found between SA111 and SA161/SA275.

### The *tst-1*-positive ST5 isolates comprised an Asia clade of virulent MRSA ST5-II

**Figure 5:**
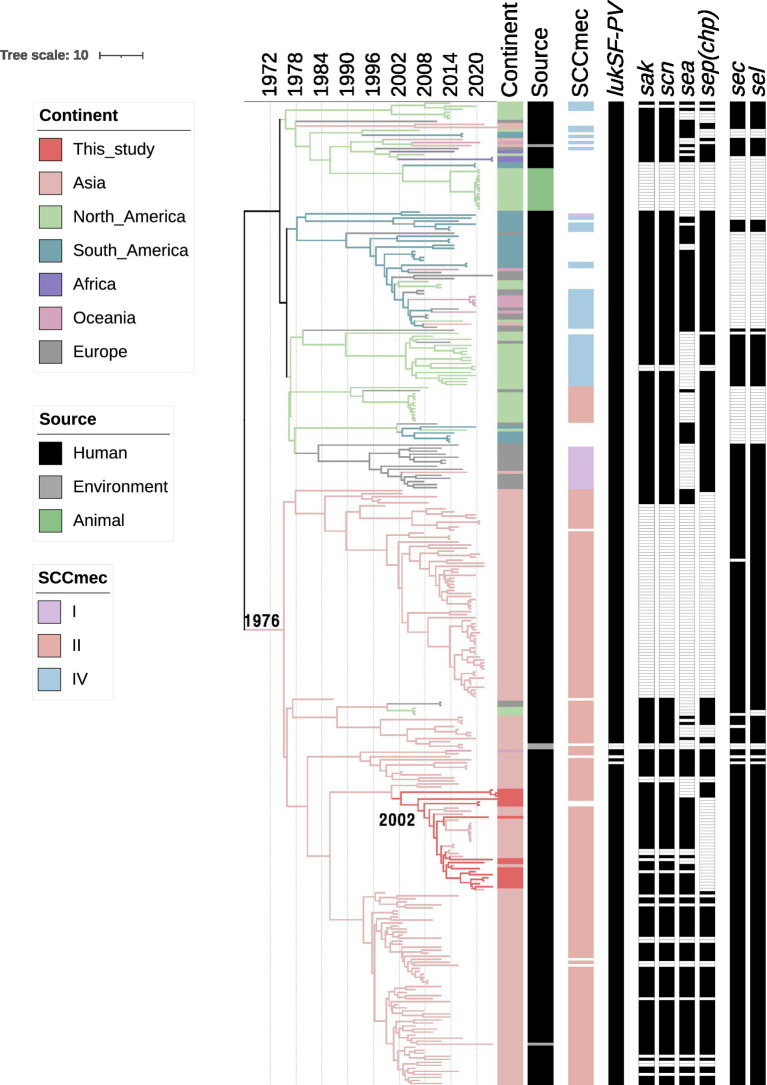
Maximum clade credibility tree of *tst-1*-positive *S. aureus* isolates.

To determine whether the prevalence of *tst-1*-positive ST5 isolates, which are strongly linked with human infections, was unique to our collection, we downloaded global ST5 isolates from the pubMLST database and constructed a temporal phylogenetic tree comprising 325 strains (Figure 5; Supplementary Table 3). Almost all *tst-1*-positive ST5 isolates (94.5%, 307/325) were from humans. Our *tst-1*-positive ST5 isolates (*n =* 16) clustered closely, and those from China, Japan, and Korea formed a distinct Asia clade. Notably, majority of isolates in this clade carried type II SCC*mec* (95.9%, 189/197) and the enterotoxin gene island SaPIs (97.5%, 192/197), although some strains lacked IEC. This Asia clade was estimated to have emerged in 1976. Ono the other hand, relatively few isolates in the non-Asia clade were MRSA or harbored the *sec* and *sel* genes. Among the non-Asia MRSA strains, the predominant SCC*mec* types were IV and I.

The tree was constructed based on the whole-genome sequencing data of 325 isolates. Source of isolation, continent, SCC*mec* types, and virulence factors are shown. The timeline of emergence of the Asia clade and subclade of the strains in this study are indicated.

## Discussion

Given the dynamic nature of *S. aureus* clonal shifts, ongoing surveillance of epidemiological trends and characterization of phylogenetic relationships among circulating strains are essential to optimize infection control protocols and constrain transmission of high-risk lineages. Clarifying the genomic and phenotypic traits of prevalent lineages is fundamental to such surveillance efforts. In this study, we performed a longitudinal genomic and phenotypic analysis of clinical *S. aureus* isolates collected over a six-year period (2018–2023). Our findings delineated the molecular and phenotypic profiles of two predominant human-associated lineages, CC398 and ST5, and further expanded our knowledge of their genomic variations and virulence-associated characteristics.

Our epidemiological analysis further revealed that MRSA prevalence ranged from 25 to 50%, consistent with previous findings in China (Lee et al., 2018). The most frequently identified MRSA clones were ST239-III (3A), ST398-V, ST59-IV (2B), and ST5-II (2A). Notably, the two clones that were prevalent in our collection [i.e., CC5/ST5 and CC8 (ST239)] have been previously reported as major causes of bloodstream infections in China and Japan (Souza et al., 2024; Turner et al., 2019), underscoring their clinical significance across regions (Souza et al., 2024; Turner et al., 2019).

Compared with the CC8 MRSA clone previously reported in the United States (USA300/500, ST8-IV), our CC8 clone was predominantly characterized by ST239-III (3A) rather than ST8-IV (Souza et al., 2024). In the 2000s, the ST239-III lineage has been reported to cause nosocomial MRSA infections in most hospitals in Asia (Smyth et al., 2010). In addition, the distinct MDR pattern exhibited by the ST239-III (3A) clone in this study may be associated with its widespread dissemination.

Meanwhile, ST398 is usually associated with livestock infection in pigs and humans exposed to animals (Fernandez et al., 2024). Heavy use of the broad-spectrum antibiotics, such as tetracycline and *β*-lactams, in veterinary medicine has led to acquisition of ARGs, such as *tetM*, type IV SCC*mec* in horse-associated CC398, and short type V SCC*mec* in pig-associated CC398 (Murray et al., 2022). In our collection, only a fraction of CC/ST398 strains carried *tetM*, and nearly all exhibited *chp*-*sak*-*scn*, which encoded the type B IEC-positive pattern, a distinct characteristic of human-associated CC398 clade (Fernandez et al., 2024). Moreover, all SCC*mec* elements in the CC/ST398 isolates in this study were long structural variants, which could not be accurately identified using short-read sequencing data; this further indicated human-adapted genetic features and suggested that this lineage has evolved to successfully colonize humans and persist as an important pathogen in clinical settings (He et al., 2018). Our observation on the rising trend for ST398 from 2019 to 2023 aligned with the broader epidemiological shift of *S. aureus* clones in China. A national multicenter longitudinal study reported that the percentage of CC398 in MRSA isolates increased from nearly undetectable levels in 2014–2015 to 8.9% in 2020 (Wang et al., 2022). In Shanghai, long-term monitoring revealed that ST398 has become one of the most dominant clones in pediatric infections since 2017, reaching a prevalence that was comparable with that of the previously predominant ST59 (Pan et al., 2025). Collectively, these epidemiological and genomic observations suggested that ST398, which was originally a livestock-associated lineage, displays genomic signatures consistent with human adaptation and has been increasing in circulation in clinical settings across China. These findings highlighted the need for continued molecular surveillance.

The CC5/ST5 clone in this study comprised a special subclone ST5-II (2A), which was positive for *tst-1* and IEC and showed enhanced virulence, based on their MDR and phenotypic characteristics *in vitro* (Jian et al., 2021). This clade has been previously identified within the CC5/USA100 MRSA lineage, which is responsible for life-threatening infections, and is recognized for its robust capability for biofilm formation (Lee et al., 2018). Accordingly, all 14 ST5-II (2A) isolates harbored a SaPI carrying *tst-1*, *sec*, and *sel* genes, and most of them exhibited active biofilm formation capabilities (Murdoch et al., 2009). In contrast, of the 16 ST5 MSSA isolates in our collection, only 2 were positive for *tst-1* and 3 were positive for *sec*. Phenotypic tests for ST5 MSSA strains of SA56 and SA231 revealed a potent cell lysis capability alongside a diminished biofilm formation capacity. Notably, our study revealed that cocarriage of *tst-1* and *sec* was associated with two different mobile genetic elements: *tst-1*, *sec*, and *sel* genes were carried by the SaPI, whereas EC was maintained on a phiN315-like *hlb*-converting phage (Rohmer and Wolz, 2021). Phylogeographic analysis indicated that the *tst-1*-positive ST5-II (2A) clade in this study clustered closely with the reported Asian ST5 MRSA group, with features of harboring hiN315-like *hlb*-converting phage, enterotoxin gene island, type II SCC*mec* elements, and the *tst-1* gene (Chen et al., 2024).

Previous studies on the large chromosome inversion of *S. aureus* have indicated that this genomic variation could lead to phenotype changes in colony morphology, hemolytic activity, and cell invasion capability (Guerillot et al., 2019; Cui et al., 2012). However, no distinct differences in chromosomal rearrangement were observed among the ST5-II (2A) strains of SA111, SA161, and SA275 in this study. Moreover, despite the absence of chromosomal inversion differences between SA161 and SA275, SA161 exhibited stronger hemolytic activity due to the complete *hlb* gene in its genome. Therefore, we speculated that the difference may be attributed to the distinct genomic background of the strains (Taki et al., 2022).

This study had several limitations. First, although the sampling framework used in this study might theoretically influence interpretation of population structure among different specimen types, our bioinformatic analyses did not identify significant genomic differences among the isolates from different anatomical sources. Importantly, although the major STs showed distinct distributions across infection sites, the isolation source itself was not associated with substantial genomic divergence. This observation was further supported by recent pangenome studies, which indicated that ST is the primary driver of *S. aureus* genomic plasticity, and isolation source only played a secondary role (Liu et al., 2022). Second, the sample size in this single-center study may be limited. Nevertheless, the strong alignment of our findings with those from larger-scale, multicenter studies (Souza et al., 2024; Turner et al., 2019) enhanced the credibility and suggested broader relevance of our conclusions.

Population-based, multiyear genomic surveillance is crucial for deciphering the epidemiological and genetic features of *S. aureus*. In this longitudinal surveillance study, we identified prevalent lineages, including the virulent ST5-II MRSA, which is genetically related with circulating strains in Asia; MDR CC8 (ST239); and the human-adapted CC398 lineage. Our findings provided valuable insights into the genomic and phenotypic profiles associated with antimicrobial resistance, immune evasion, and transmission-related features and can help in the interpretation of the high prevalence of these lineages among clinical *S. aureus* isolates.

This study performed phenotypic and molecular characterization of clinical *S. aureus* isolates collected at our hospital over six consecutive years. National antimicrobial resistance surveillance programmes mainly report overall resistance rates, while our molecular data uncovered distinct epidemiological characteristics of local strains. Based on these findings, we have optimised hospital infection prevention and control (IPC) protocols, strengthened targeted surveillance in high-risk areas including ICU, surgical wards and burn wards, standardised isolation precautions, and guided rational antimicrobial use. This single-centre molecular epidemiological investigation provides solid evidence for developing tailored IPC strategies and preventing nosocomial spread of drug-resistant staphylococci.

## Data Availability

The datasets presented in this study can be found in online repositories. The names of the repository/repositories and accession number(s) can be found at: https://db.cngb.org/cnsa/project/CNP0006765_44ef2d41/reviewlink/, CNP0006765.

## Footnotes

1
https://pubmlst.org/

2
https://www.phyloviz.net/

3
https://cge.food.dtu.dk/services/SCCmecFinder/

4
https://github.com/mjsull/spa_typing

5
https://github.com/tseemann/snippy

6
https://itol.embl.de/
